# Combination of sugar-sweetened beverage consumption, screen-based sedentary time, and sleep duration and their association with South Korean adolescents' mental health

**DOI:** 10.3389/fpubh.2023.1293505

**Published:** 2024-01-19

**Authors:** Jin Suk Ra

**Affiliations:** College of Nursing, Chungnam National University, Daejeon, Republic of Korea

**Keywords:** adolescent, sugar-sweetened beverages, screen time, sleep duration, mental health

## Abstract

**Introduction:**

This study examines the combinations of sugar-sweetened beverage (SSB) consumption, screen-based sedentary time, and sleep duration and their association with adolescents' depressive symptoms and suicidal ideation.

**Methods:**

This research followed a crosssectional study design. Secondary data analysis was conducted on the data collected from 21,046 high school students who had participated in the 17th Korea Youth Risk Behavior Web-Based Survey in 2021. A complex sampling analysis, including descriptive and logistic regression analyses, was conducted in SPSS Statistics 26.0.

**Results:**

The combination of low SSB consumption, excessive screen-based sedentary time, and short sleep duration was associated with an increase in depressive symptoms. In addition, a combination of medium/high SSB consumption, appropriate/excessive screen-based sedentary time, and short sleep duration was associated with an increase in depressive symptoms. Finally, the combination of high SSB consumption, excessive screen-based sedentary time, and short sleep duration was associated with an increase in suicidal ideation.

**Discussion:**

The findings demonstrate that healthcare providers should develop and conduct family-and school-based programs to restrict SSB consumption, screen-based sedentary behaviors, and sleep duration to improve adolescents' mental health.

## 1 Introduction

Depressive symptoms are a common mental health problem that develops in adolescence and results in suicidal ideation and behaviors (1, 2). According to a systematic review and meta-analysis, the global prevalence of depressive symptoms among adolescents increased by 34% between 2001 and 2020 (3). In addition, the prevalence of depressive symptoms among South Korean adolescents increased continuously from 27.8% in 2015 to 34.6% in 2019 (4). These symptoms may be related to suicidal ideation, a major cause of early death in adolescence (5). Early identification focused on modifiable associated factors, using tools such as the transdiagnostic model, is important for the effective prevention of mental health problems (6). For example, using the transdiagnostic model for prevention in youth mental health, Colizzi et al. (7) reported the need to identify risk and protective factors that modulate gene expression and stress response associated with mental health status. Hence, the identification of modifiable risk factors is necessary to recognize adolescents with severe depressive symptoms and suicidal ideation.

Adolescence is an important stage for the development of healthy lifestyle behaviors (e.g., diet, activity, and sleep) associated with mental health (8, 9). However, many adolescents have unhealthy dietary habits, engage in insufficient activity (e.g., prolonged screen-based sedentary behaviors), and experience short sleep duration (10–12). Adolescents in high school tend to consume sugar-sweetened beverages (SSBs), such as soda, more frequently than individuals in other developmental stages. According to Southerland et al. (13), ~63% of American adolescents consume SSBs more than once daily. In addition, ~96% of South Korean high school students routinely consume SSBs (14), and 40% of South Korean high school students consume SSBs more than once daily. While SSB consumption is a habitual dietary behavior of adolescents, higher SSB consumption is negatively associated with mental health in high school students, including an increase in depressive symptoms and suicidal ideation (15, 16).

Currently, most adolescents have exposure and access to various screens (e.g., television, computer, tablet, and smartphone screens) (17). The recommended screen time for leisure activities is < 2 h/day for children and adolescents in the United States (18), and 32.9% of US children and adolescents aged 6–17 years old satisfy this recommendation (19). According to a population-based study on adolescents in the US, the average screen-based sedentary time of adolescents aged 14–17 years was 4 h and 35 min a day, which is the longest reported screen-based sedentary time among children and adolescents (20). In addition, according to a national study of South Korean middle and high school students, the screen-based sedentary time of 66.5% of adolescents was ≥2 h/day (21). Increased screen-based sedentary time was associated with poor mental health including depressive symptoms and suicidal ideation among high school students (22, 23).

Among high school students, short sleep duration is a common lifestyle behavior (11). Although the American Academy of Sleep Medicine recommends 8–10 h of sleep a day for adolescents aged 13–17 years (24), ~69% of high school students sleep < 7 h/night (25). The average sleep duration of South Korean high school was 6.2 h/night on weekdays (4). Short durations of sleep are associated with mental health problems (e.g., poor mood regulation, depressive symptoms, and suicidal ideation) (23, 26, 27). Additionally, SSB consumption, screen-based sedentary time, and short sleep duration might be correlated. Prolonged screen-based sedentary time is associated with increased SSB consumption and short sleep duration in adolescents (28, 29). In addition, short sleep duration and increased SSB consumption are interrelated (30, 31). Hence, they might form clustered lifestyle behaviors, which have neutralizing or synergistic effects in combination (32). In this context, associations between the combination of SSB consumption, screen-based sedentary time, and short sleep duration and mental health (particularly depressive symptoms and suicidal ideation) may differ from the association between each of these variables and mental health. However, few studies have examined the associations between the combination of these variables and adolescents' mental health. Therefore, this study examined the combinations of SSB consumption, screen-based sedentary time, and sleep duration and their associations with high school students' depressive symptoms and suicidal ideation.

The study adjusted for covariates identified following a literature review of the factors associated with adolescents' depressive symptoms and suicidal ideation based on a biopsychosocial model, which focuses on the impact of biological (e.g., sex), social (e.g., socioeconomic status), and psychological (e.g., behaviors) factors on individual health (33). According to Engert et al. (34), mental health is influenced by interactions between biological and psychosocial characteristics. Further, Porter (35) emphasized that various mental health-associated factors should be considered as related to the biopsychosocial aspects. A literature review found sex to be biologically associated with depressive symptoms and suicidal ideation (36, 37). Further, grade, academic achievements, and socioeconomic status are socially associated with depressive symptoms and suicidal ideation (37, 38). Among the psychological factors, those associated with depressive symptoms and suicidal ideation include perceived health status (38), perceived body shape (39), skipping breakfast (40), fast-food consumption (14), moderate and vigorous physical activity (38), current cigarette consumption (37, 38), current alcohol consumption (37, 38), sexual intercourse experience (38), and habitual substance use experience (37). Hence, the purpose of this study was to identify the combinations of SSB consumption, screen-based sedentary time, and sleep duration and their associations with adolescents' depressive symptoms and suicidal ideation among South Korean high school students while adjusting for covariates.

## 2 Materials and methods

### 2.1 Design and sample

A cross-sectional study design was used to conduct a secondary analysis of data from the 17th Korea Youth Risk Behavior Web-based Survey (KYRBS), which is a national survey of the health status and its associated factors (e.g., behaviors and environmental characteristics) of South Korean adolescents; that is, middle and high school students. Among the 59,066 adolescents (30,015 middle school students and 27,885 high school students) from 800 schools across 17 South Korean provinces, 54,848 adolescents (92.9%), comprising 30,015 middle school students (96.3%) and 24,833 high school students (89.1%), participated in the KYRBS. The data of 21,046 high school students (10,812 boys and 10,234 girls) were analyzed after excluding all middle school students and 3,787 high school students with missing data on SSB consumption, screen-based sedentary time on weekdays and weekends, wake-up time and bedtime on weekdays and weekends, and covariates.

### 2.2 Measurements

#### 2.2.1 Outcome variables

##### 2.2.1.1 Depressive symptoms

A single “yes” or “no” question asking whether they had felt sadness or hopelessness in the past 12 months was used to assess the participants' depressive symptoms.

##### 2.2.1.2 Suicidal ideation

A single “yes” or “no” question asking whether they had seriously considered suicide in the past 12 months was used to assess the participants' suicidal ideation.

#### 2.2.2 Independent variables

##### 2.2.2.1 Sugar-sweetened beverage consumption

Two questions regarding the participants' consumption of soda and other beverages with added sugar in the past 7 days were used to assess SSB consumption. For each item, the SSB consumption frequency was calculated as the number of times a week (e.g., 3–4 times a week = 3.5 times a week and once daily = 7 times a week) according to a response scale. Subsequently, the calculated SSB consumption frequency values were summed. Finally, the summed values of SSB consumption frequency were grouped into first (Q1), second (Q2), and third (Q3) quartiles of low, medium, and high consumption, respectively.

##### 2.2.2.2 Screen-based sedentary time

The participants were asked a single question about their average daily duration of screen-based sedentary behaviors (e.g., smartphone use and video games) on weekdays and weekends to assess their screen-based sedentary time. The average daily screen-based sedentary time was classified into ≥2 h and < 2 h. According to the recommendations of the American Academy of Pediatrics Committee on Public Education (18), screen-based sedentary duration ≥2 h/day is excessive, and a duration < 2 h/day is appropriate.

##### 2.2.2.3 Sleep duration

The participants were asked four questions regarding bedtime and wake-up time on weekdays and weekends to assess their sleep duration. Based on these bedtimes and wake-up times, the average daily sleep duration (hours/day) was calculated. According to the recommendations of the American Academy of Sleep Medicine (24), a sleep duration < 8 h/day is short, and a duration ≥8 h/day is sufficient.

#### 2.2.3 Covariates

Table 1 presents the questions used to assess the biological, social, and psychological factors of covariates and the response scale based on the questions.

**Table 1:** Variables and measurement of covariates.

**Variables**	**Measurement**
**Biological factors**	
Gender	Boy or girl
**Social factors**	
Grade	1st, 2nd, and 3rd
Academic achievement	Assessed subjective academic achievements with a single question. Answers were classified into high, middle, or low.
Family's socioeconomic status	Assessed subjective socioeconomic status of family with a single question. Answers were classified into high, middle, or low.
**Psychological factors**	
Perceived health status	Assessed subjective health status with a single question. Answers were classified into healthy, fair, or unhealthy.
Perceived body shape	Assessed subjective body shape with a single question. Answers were classified into fat, average (not fat and not thin), or thin.
Skipping breakfast	Assessed days of eating breakfasts in a week with a single question. Answers were classified into yes (with days of skipping breakfast) or no (without days of skipping breakfast).
Fast-food consumption	Assessed frequency of fast-food consumption in a week with a single question. Frequency of fast-food consumption was converted to times a week, and this was finally categorized into three quantile groups: first quartile (Q1) = low consumption, second quartile (Q2) = medium consumption, and third quartile (Q3) = high consumption.
Moderate and vigorous physical activity	Assessed frequency (number of days) for moderate and vigorous physical activity in a week with two questions. Answers were classified into ≥ 3 days or < 3 days a week based on the recommendations for physical activity guidelines for children in the US and Korea (40).
Current cigarette consumption	Assessed smoking experiences (days) in a month (30 days) with a single question. Answers were classified into yes or no.
Current alcohol consumption	Assessed alcohol consumption experiences (days) in a month (30 days) with a single question. Answers were classified into yes or no.
Experience of sexual intercourse	Assessed experiences of sexual intercourse with a single question. Answers were classified into yes or no.
Experience of substance use	Assessed experiences of habitual substance use except for therapeutic purposes. Answers were classified into yes or no.

### 2.3 Ethical considerations

The Institutional Review Board approved this study's exemption from review because the study used secondary data from the 17th KYRBS (approval no.: 202305-SB-075-01).

### 2.4 Statistical analysis

A complex sampling analysis, including descriptive and logistic regression analyses, was conducted in SPSS Statistics 26.0 (IBM, Armonk, NY) according to the analysis guidelines of the 17th KYRBS. The frequencies and percentages of depressive symptoms, suicidal ideation, SSB consumption, screen-based sedentary time, sleep duration, and covariates were analyzed using descriptive statistics. Further, a logistic regression analysis was performed to clarify the combinations of SSB consumption, screen-based sedentary time, and sleep duration and their associations with adolescents' depressive symptoms and suicidal ideation while adjusting for covariates (model 1, 2, and 3).

## 3 Results

### 3.1 Mental health experience and lifestyle behaviors

In this study, 27.7% of participants experienced depressive symptoms in the past 12 months. In addition, 11.3% reported experiencing suicidal ideation in the past 12 months (Table 2). Regarding SSB consumption, the participants were classified into three quartile groups: 37.3% in Q1 (low), 26.3% in Q2 (medium), and 36.4% in Q3 (high). In addition, 70.7% of the participants recorded excessive (≥2 h/day) screen-based sedentary time. Regarding sleep duration, 84.5% of the participants had short sleep duration (< 8 h/day; Table 2). Further, combined lifestyle behaviors, such as SSB consumption, screen-based sedentary time, and sleep duration, were classified into 12 groups. The combination of high (Q3) SSB consumption, excessive screen-based sedentary time, and short sleep duration had the highest prevalence (22.5%; Table 2).

**Table 2:** Mental health experience and lifestyle behaviors.

**Variables**		**Categories**	***n* (%)**
**Mental health**			
Depressive symptoms		Yes	5,582 (27.7)
		No	15,464 (72.3)
Suicidal ideation		Yes	2,397 (11.3)
		No	18,649 (88.7)
**Independent lifestyle behaviors**			
Sugar-sweetened beverage consumption		Q1 (low)	7,886 (37.3)
		Q2 (medium)	5,498 (26.3)
		Q3 (high)	7,662 (36.4)
Screen-based sedentary behaviors (hours a day)		< 2	6,121 (29.3)
		≥2	14,925 (70.7)
Sleep duration (hours a day)		≥8	3,409 (15.5)
		< 8	17,637 (84.5)
**Combined lifestyle behaviors**			
Sugar-sweetened beverage consumption	Screen based sedentary behaviors (hours a day)	Sleep duration (hours a day)	
Q1 (low)	< 2	≥8	440 (2.0)
	< 2	< 8	2,021 (9.7)
	≥2	≥8	1,004 (4.7)
	≥2	< 8	4,421 (20.9)
Q2 (medium)	< 2	≥8	194 (0.9)
	< 2	< 8	1,392 (6.8)
	≥2	≥8	607 (2.7)
	≥2	< 8	3,305 (16.0)
Q3 (high)	< 2	≥8	284 (1.3)
	< 2	< 8	1,790 (8.6)
	≥2	≥8	880 (3.9)
	≥2	< 8	4,708 (22.5)
Covariates			
**Biological factors**			
Sex		Boys	10,812 (51.6)
		Girls	10,234 (48.4)
**Social factors**			
Grade		1st	7,269 (32.3)
		2nd	7,284 (33.6)
		3rd	6,493 (34.1)
Academic achievement		Low	1,929 (9.1)
		Middle	16,795 (79.9)
		High	2,322 (11.0)
Family's socioeconomic status		Low	513 (2.3)
		Middle	18,868 (89.7)
		High	1,665 (8.0)
**Psychological factors**			
Perceived health status		Unhealthy	2,157 (10.4)
		Fair	5,555 (26.2)
		Healthy	13,334 (63.4)
Perceived body shape		Being fat	8,495 (40.0)
		In average	7,502 (35.6)
		Skinny	5,049 (24.4)
Skipping breakfast		Yes	16,388 (77.8)
		No	4,658 (22.2)
Fast food consumption		Q1 (low)	15,347 (72.7)
		Q2 (medium)	4,602 (22.0)
		Q3 (high)	1,097 (5.3)
Moderate and vigorous physical activity (a week)		≥3 days	7,297 (34.2)
		< 3 days	13,749 (65.8)
Current cigarette consumption		Yes	1,363 (6.2)
		No	19,683 (93.8)
Current alcohol consumption		Yes	3,201 (14.9)
		No	17,845 (85.1)
Experience of sexual intercourse		Yes	2,116 (8.5)
		No	22,717 (91.5)
Experience of habitual substance use		Yes	114 (0.6)
		No	20,902 (99.4)

N = 21,046.

n, unweighted; %, weighted.

Q1, first quantile; Q2, second quantile; Q3, third quantile.

### 3.2 Association between independent lifestyle behavior and adolescents' mental health

In Model 1, which included adjusted biological factors, medium [Q2; adjusted odds ratio (AOR) = 1.15, 95% confidence interval (CI) = 1.05–1.26] and high (Q3; AOR = 1.41, 95% CI = 1.29–1.53) SSB consumption were associated with greater depressive symptoms than low SSB consumption (Q1; Table 3). High SSB consumption was also associated with higher suicidal ideation than low SSB consumption. Excessive screen-based sedentary time was associated with greater depressive symptoms (AOR = 1.62, 95% CI = 1.51–1.75) and higher suicidal ideation (AOR = 1.17, 95% CI = 1.06–1.30) than the recommended appropriate time (< 2 h daily; Table 3). Short sleep duration was associated with greater depressive symptoms (AOR = 1.59, 95% CI = 1.48–1.72) and higher suicidal ideation (AOR = 1.81, 95% CI = 1.62–2.03) than the recommended sufficient time (≥8 h/day; Table 3).

**Table 3:** Association between independent lifestyle behavior and adolescents' mental health.

**Independent lifestyle behaviors**		**Model 1**		**Model 2**		**Model 3**	
		**Depressive symptoms**	**Suicidal ideation**	**Depressive symptoms**	**Suicidal ideation**	**Depressive symptoms**	**Suicidal ideation**
		**Adjusted odds ratio (95% confidence interval)**		**Adjusted odds ratio (95% confidence interval)**		**Adjusted odds ratio (95% confidence interval)**	
Sugar-sweetened beverage consumption	Q1 (low)	1.00	1.00	1.00	1.00	1.00	1.00
	Q2 (medium)	1.15 (1.05–1.26)^*^	1.09 (0.95–1.24)	1.15 (1.05–1.26)^*^	1.09 (0.95–1.24)	1.10 (0.99–1.12)	1.03 (0.90–1.18)
	Q3 (high)	1.41 (1.29–1.53)^*^	1.30 (1.18–1.44)^*^	1.39 (1.28–1.51)^*^	1.28 (1.16–1.42)^*^	1.18 (1.08–1.30)^*^	1.10 (0.98–1.23)
Screen based sedentary behaviors (hours a day)	< 2	1.00	1.00	1.00	1.00	1.00	1.00
	≥2	1.62 (1.51–1.75)^*^	1.17 (1.06–1.30)^*^	1.05 (0.98–1.13)	1.14 (1.03–1.26)^*^	1.02 (0.94–1.10)	1.11 (1.0–1.24)^*^
Sleep duration (hours a day)	≥8	1.00	1.00	1.00	1.00	1.00	1.00
	< 8	1.59 (1.48–1.72)^*^	1.81 (1.62–2.03)^*^	1.32 (1.20–1.44)^*^	1.37 (1.18–1.59)^*^	1.26 (1.15–1.38)^*^	1.28 (1.10–1.49)^*^

Model 1 = Adjusted for biological factors associated with adolescents' depressive symptoms and suicidal ideation.

Model 2 = Adjusted for biological and social factors associated with adolescents' depressive symptoms and suicidal ideation.

Model 3 = Adjusted for biological, social, and psychological factors associated with adolescents' depressive symptoms and suicidal ideation.

* p < 0.05.

Q1, first quantile (reference); Q2, second quantile; Q3, third quantile.

In Model 2, which adjusted biological and social factors, high SSB consumption was associated with greater depressive symptoms (AOR = 1.39, 95% CI = 1.28–1.51) and higher suicidal ideation (AOR = 1.28, 95% CI = 1.16–1.42) than low SSB consumption (Q1; Table 3). In addition, excessive screen-based sedentary time was associated with higher suicidal ideation (AOR = 1.14, 95% CI = 1.03–1.26) than the recommended appropriate time (< 2 h daily; Table 3). Short sleep duration was associated with greater depressive symptoms (AOR = 1.32, 95% CI = 1.20–1.44) and higher suicidal ideation (AOR = 1.37, 95% CI = 1.18–1.59) than the recommended sufficient time (≥8 h/day; Table 3).

In Model 3, which adjusted biological, social, and psychological factors, high SSB consumption was associated with greater depressive symptoms (AOR = 1.18, 95% CI = 1.08–1.30) than low SSB consumption (Q1; Table 3). However, SSB consumption was not associated with suicidal ideation. In addition, excessive screen-based sedentary time was associated with higher suicidal ideation (AOR = 1.11, 95% CI = 1.00–1.24) than the recommended appropriate time (< 2 h daily), whereas screen-based sedentary time was not associated with depressive symptoms (Table 3). Further, short sleep duration was associated with greater depressive symptoms (AOR = 1.26, 95% CI = 1.15–1.38) and higher suicidal ideation (AOR = 1.28, 95% CI = 1.10–1.49) than the recommended sufficient time (≥8 h/day; Table 3).

### 3.3 Association between combined lifestyle behaviors and adolescents' mental health

The combination of low SSB consumption, appropriate screen-based sedentary time, and sufficient sleep duration was used as a reference group. In Model 1, the combination of low SSB consumption, excessive screen-based sedentary time, and short sleep duration was associated with greater depressive symptoms (AOR = 1.51, 95% CI = 1.13–2.03) and higher suicidal ideation (AOR = 1.61, 95% CI = 1.07–2.42) than the reference group. In addition, the combination of medium SSB consumption, appropriate screen-based sedentary time, and short sleep duration was associated with greater depressive symptoms (AOR = 1.55, 95% CI = 1.14–2.12) and higher suicidal ideation (AOR = 1.60, 95% CI = 1.04–2.48) than the reference group. The combination of medium SSB consumption, excessive screen-based sedentary time, and short sleep duration was associated with greater depressive symptoms (AOR = 1.60, 95% CI = 1.19–2.15) and higher suicidal ideation (AOR = 1.69, 95% CI = 1.12–2.55) than the reference group. The combination of high SSB consumption, appropriate screen-based sedentary time, and sufficient sleep duration was associated with greater depressive symptoms (AOR = 1.64, 95% CI = 1.08–2.48) than the reference group. The combination of high SSB consumption, appropriate screen-based sedentary time, and short sleep duration was associated with greater depressive symptoms (AOR = 1.94, 95% CI = 1.42–2.65) and higher suicidal ideation (AOR = 1.70, 95% CI = 1.13–2.55) than the reference group. The combination of high SSB consumption, excessive screen-based sedentary time, and sufficient sleep duration was associated with greater depressive symptoms (AOR = 1.68, 95% CI = 1.19–2.37) than the reference group. The combination of high SSB consumption, excessive screen-based sedentary time, and short sleep duration was associated with greater depressive symptoms (AOR = 1.91, 95% CI = 1.43–2.56) and higher suicidal ideation (AOR = 2.10, 95% CI = 1.42–3.11) than the reference group (Tables 4, 5).

**Table 4:**
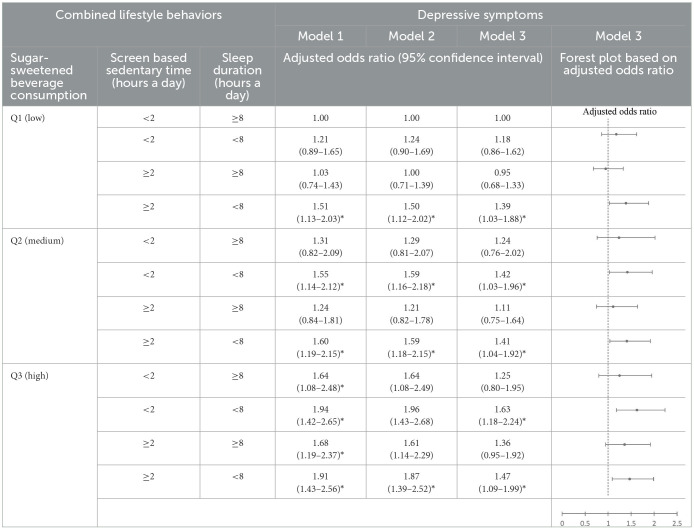
Association between combined lifestyle behaviors and adolescents' depressive symptoms.

Model 1 = Adjusted for biological factors associated with depressive symptoms and suicidal ideation in adolescents.

Model 2 = Adjusted for biological and social factors associated with depressive symptoms and suicidal ideation in adolescents.

Model 3 = Adjusted for biological, social, and psychological factors associated with depressive symptoms and suicidal ideation in adolescents.

^*^p < 0.05.

Q1, first quantile (reference); Q2, second quantile; Q3, third quantile.

**Table 5:**
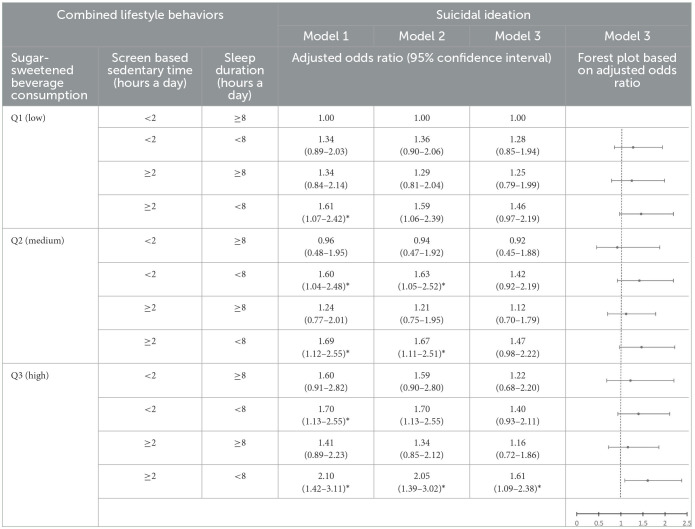
Association between combined lifestyle behaviors and adolescents' suicidal ideation.

Model 1 = Adjusted for biological factors associated with depressive symptoms and suicidal ideation in adolescents.

Model 2 = Adjusted for biological and social factors associated with depressive symptoms and suicidal ideation in adolescents.

Model 3 = Adjusted for biological, social, and psychological factors associated with depressive symptoms and suicidal ideation in adolescents.

^*^p < 0.05.

Q1, first quantile (reference); Q2, second quantile; Q3, third quantile.

In Model 2, the combination of low SSB consumption, excessive screen-based sedentary time, and short sleep duration was associated with greater depressive symptoms (AOR = 1.50, 95% CI = 1.12–2.02) than the reference group. In addition, the combination of medium SSB consumption, appropriate screen-based sedentary time, and short sleep duration was associated with greater depressive symptoms (AOR = 1.59, 95% CI = 1.16–2.18) and higher suicidal ideation (AOR = 1.63, 95% CI = 1.05–2.52) than the reference group. The combination of medium SSB consumption, excessive screen-based sedentary time, and short sleep duration was associated with greater depressive symptoms (AOR = 1.59, 95% CI = 1.18–2.15) and higher suicidal ideation (AOR = 1.67, 95% CI = 1.11–2.51) than the reference group. The combination of high SSB consumption, excessive screen-based sedentary time, and short sleep duration was associated with greater depressive symptoms (AOR = 1.87, 95% CI = 1.39–2.52) and higher suicidal ideation (AOR = 2.05, 95% CI = 1.39–3.02) than the reference (Tables 4, 5).

In Model 3, the combination of low SSB consumption, excessive screen-based sedentary time, and short sleep duration was associated with greater depressive symptoms (AOR = 1.39, 95% CI = 1.03–1.88) than the reference group. In addition, the combination of medium SSB consumption, appropriate screen-based sedentary time, and short sleep duration (AOR = 1.42, 95% CI = 1.03–1.96) and the combination of medium SSB consumption, excessive screen-based sedentary time, and short sleep duration were associated with greater depressive symptoms (AOR = 1.41, 95% CI = 1.04–1.92) than the reference group. Finally, the combination of high SSB consumption, appropriate screen-based sedentary time, and short sleep duration (AOR = 1.63, 95% CI = 1.18–2.24) and the combination of high SSB, excessive screen-based sedentary time, and short sleep duration were associated with greater depressive symptoms (AOR = 1.47, 95% CI = 1.09–1.99) than the reference (Tables 4, 5).

## 4 Discussion

This study identified the combinations of SSB consumption, screen-based sedentary time, and sleep duration and their associations with depressive symptoms and suicidal ideation among South Korean high school students after controlling for the covariates (biological, social, and psychological factors). Based on transdiagnostic approaches to mental health, signs and symptoms of mental health emerge from the interaction of underlying multi-factors, including biological, behavioral, psychosocial, and socio-cultural factors (41). Thus, an identification of underlying modifiable vulnerable factors of the target population might be the first step to developing preventive strategies for mental health problems (6). In these contexts, considering the interaction between lifestyle behavior and covariates, identifying significant lifestyle behaviors associated with adolescents' depressive symptoms and suicidal ideation can primarily require processes for prevention and relieving depressive symptoms and suicidal ideation in adolescence.

The results revealed that a combination of low SSB consumption, excessive screen-based sedentary time, and short sleep duration was associated with depressive symptoms. Additionally, a combination of medium/high SSB consumption, appropriate/excessive screen-based sedentary time, and short sleep duration was associated with an increase in depressive symptoms. Therefore, from low to high SSB consumption, combined excessive screen-based sedentary time and short sleep duration may be associated with increased depressive symptoms. In addition, even when screen-based sedentary time is appropriate, a combination of medium/high SSB consumption and short sleep duration is associated with increased depressive symptoms. Finally, a combination of high SSB consumption, excessive screen-based sedentary time, and short sleep duration is associated with increased suicidal ideation.

In earlier studies, frequent SSB consumption, prolonged screen-based sedentary time, and short sleep duration were associated with depressive symptoms and suicidal ideation (15, 36, 42). According to Ra (15), excessive SSB consumption is associated with increased depressive symptoms and suicidal ideation in adolescents. Similarly, SSB consumption more than once a day was associated with a 2.28-fold increase in Chinese adolescents' depressive symptoms (43). In a study of adolescents from 32 countries, SSB consumption more than thrice a day was associated with 1.36-fold and 1.43-fold increases in suicidal attempts, respectively (44).

Yau and Potenza (45) reported that emotional distress, including depression, promotes the consumption of sweet food. Emotional distress triggers appetite and induces increased eating as a coping behavior, even in the absence of hunger (46, 47). Furthermore, emotional eating to cope with negative emotions can diminish psychological wellbeing and weaken emotion regulation, resulting in poor mental health (48). Hence, along with restricting SSB consumption, healthy coping methods for emotional distress (e.g., depressive symptoms) should be taught to adolescents to improve their mental health.

Xu et al. (49) reported that SSB consumption was an intermediary factor between screen-based sedentary time and depressive symptoms. An increase in screen-based sedentary time sequentially leads to SSB consumption and, finally, results in depressive symptoms (49). Increased screen-based sedentary time may cause frequent exposure to SSB advertisements, as well (50). In addition, adolescents addicted to smartphones, the Internet, and video games may be socially isolated, have less interpersonal communication, and avoid shared mealtimes. They may also often eat convenience/fast foods and SSBs alone while engaging in screen-based sedentary behaviors. Finally, their social isolation, weak interpersonal relationships, and lack of social activities may cause depressive symptoms in adolescents (51).

Face-to-face communication (as an in-person social interaction) is important for establishing emotional connection and intimacy, which prevent loneliness (52). In contrast, extensive electronic communication with screen-based devices (e.g., smartphones and computers) and screen-based sedentary activities, such as leisure activities, may increase loneliness and decrease emotional closeness (53). Therefore, adolescents with prolonged screen-based sedentary time may experience in-person social disconnection and low self-esteem, which may lead to depressive symptoms and suicidal ideation. According to Twenge and Campbell (20), adolescents with fewer in-person social interactions and prolonged screen-based sedentary time exhibit an increase in depressive symptoms and suicidal behaviors, including suicidal ideation. Prolonged screen-based sedentary time (≥7 h/day) is associated with a higher risk of depressive symptoms than appropriate sedentary time (1 h/day) (20). In addition, a cohort study showed that prolonged screen-based sedentary time (mean = 3.99 h/day) was associated with a 1.09-fold increase in suicidal behaviors (22). Zhang et al. (54) reported that an increase in screen-based sedentary time (mobile phone use) results in an increase in suicidal behaviors and has mediating effects on depressive symptoms. Hence, prolonged screen time is directly and indirectly (through mediation by SSB consumption) associated with increased depressive symptoms and suicidal ideation. In this context, family- and school-based intervention strategies should be developed to reduce adolescents' screen-based sedentary time. Establishing clear rules regarding screen-based media use and limiting screen time at home help reduce screen-based sedentary time (55). Ahmed et al. (56) found that engaging adolescents in physical activities during leisure time in schools reduced their screen-based sedentary time.

Furthermore, excessive screen-based sedentary time may cause sleep problems, such as a short sleep duration (28). As prolonged screen-based sedentary time affects hormones, such as cortisol and melatonin, which cause sleep disturbances (57, 58), short sleep duration is associated with a negative or depressed mood (59). Similarly, prolonged screen-based sedentary time is associated with depressive symptoms and sleep duration mediation and moderation (23, 60). Sleep deficiency may also cause biological changes, including changes in brain-derived neurotrophic factor (61), which is associated with depression (62). In addition, short sleep durations may increase SSB consumption; further, frequent SSB consumption may worsen psychological wellbeing (49, 63). Sleep deficiency adversely affects adolescents' physical and psychosocial health (e.g., it causes obesity, poor academic performance, and risky behaviors), which results in depressive symptoms (64, 65). According to an experimental study of high school students (66), adolescents who have the opportunity to sleep for 5 h reported having significantly more depressed mood and more unhappy and lethargic status than adolescents who have the opportunity to sleep for 7.5 and 10 h. A cohort study of Chinese adolescents associated short sleep durations (< 8 h/day) with depressive symptoms (67). In addition, short sleep durations of < 5 and 5–7 h/day were correspondingly associated with 2.28-fold and 1.59-fold increases in adolescents' suicidal ideation.

Further, the effects of short sleep duration on suicidal ideation were mediated by depressive symptoms (60). In these contexts, adolescents, particularly high school students, may experience an increase in depressive symptoms and suicidal ideation due to their short sleep durations. Hence, maintaining appropriate sleep durations is essential to improve adolescents' mental health. A systematic review (68) revealed the effectiveness of a combination of appropriate screen-based sedentary time and sufficient sleep duration in improving adolescents' mental health, including depressive symptoms. In another systematic review, Wilhite et al. (69) reported that the combination of excessive screen-based sedentary time and short sleep duration adversely affects adolescents' mental health. Hence, ensuring the adequacy of adolescents' sleep duration by fixing a bedtime and monitoring or limiting their screen-based media use at bedtime is important.

### 4.1 Study limitations

The results of this study contribute to the development of lifestyle modification interventions to improve adolescents' mental health. However, this study has some limitations. First, as this study involved a secondary data analysis, SSB and fast-food consumption were evaluated using the number of days of consumption rather than the quantity of consumption. Second, dependent variables (depressive symptoms and suicidal ideation) were assessed through a single self-report item. Thus, the diagnostic accuracy of mental health problems might be weak. Third, as an independent variable, SSB consumption was assessed through weekly frequency, which did not consider the total amount and calories. In addition, screen-based sedentary time and sleep duration were assessed through hours a day. However, disturbances in daily life according to screen-based sedentary time and quality of sleep must also be assessed. Thus, considering the quantity and quality of lifestyle behaviors, associations between lifestyle behaviors and mental health should be identified in further studies. Fourth, potentially associated covariates were limited to variables available from the 17th KYRBS. The outcome variables and covariates were evaluated using yes (with) or no (without) answers to a question. Future studies should evaluate these variables using instruments with appropriate validity and reliability. Fifth, the participants were South Korean adolescents, that is, high school students. Their lifestyle and health-related behaviors are likely influenced by their social and cultural environments. Future studies should confirm the current study's results for adolescents of different races and ethnicities. Finally, with the cross-sectional study design, causal relationships between independent and dependent variables could not be verified. Thus, a longitudinal study must be performed to verify causal relationships between the independent and dependent variables.

## 5 Conclusion

Results indicated that adolescents' lifestyle, along with frequent SSB consumption, prolonged screen-based sedentary time, and short sleep duration, adversely affected their mental health by worsening depressive symptoms and suicidal ideation. Therefore, family- and school-based programs aimed at changing behaviors to reduce SSB consumption, excessive screen-based sedentary behaviors, and short sleep duration should be developed.

## Data availability statement

Publicly available datasets were analyzed in this study. The data set for this study is data from the 17th Korea Youth Risk Behavior Web-based Survey (2021) conducted Korea Disease Control and Prevention Agency and can be found online by following the academic research material application procedure (https://www.kdca.go.kr/yhs).

## Ethics statement

The studies involving humans were approved by the Institutional Review Board of Chungnam National University approved this study's exemption from review because the study used secondary data from the 17th KYRBS (approval no.: 202305-SB-075-01). The studies were conducted in accordance with the local legislation and institutional requirements. Written informed consent for participation was not required from the participants or the participants' legal guardians/next of kin in accordance with the national legislation and institutional requirements.

## Author contributions

JR: Conceptualization, Formal analysis, Funding acquisition, Investigation, Methodology, Writing – original draft.
